# Editorial Note: Optimal path selection and secured data transmission in underwater acoustic sensor networks: LSTM-based energy prediction

**DOI:** 10.1371/journal.pone.0327407

**Published:** 2025-07-02

**Authors:** The PLOS One Editors

The *PLOS One* Editors issue this Editorial Note to inform readers that there was a potential competing interest between the authors and one or more people involved in peer review. After reviewing this matter, PLOS concluded that the article’s publication is supported based on expert input that was not affected by the concerns.

In addition, the following issues were noted:

- The article cited in [1] as Reference 14 was retracted after [1] was published.
- The data underlying the results were not provided with the article or its Supporting Information, contrary to what is stated in the Data Availability statement. The authors provide the underlying data in S1 File with this notice.
- The source code underlying the research reported in [1] was not provided with the article as encouraged by PLOS policy on Sharing Code. The authors provided the code in post-publication discussions and agreed to make the code available to readers on request.

## Supporting information

S1 File Underlying data. (XLSX)
